# Large-Scale Monitoring of the Frequency of Ryanodine Receptor Target-Site Mutations Conferring Diamide Resistance in Brazilian Field Populations of Fall Armyworm, *Spodoptera frugiperda* (Lepidoptera: Noctuidae)

**DOI:** 10.3390/insects13070626

**Published:** 2022-07-13

**Authors:** Daniela M. Okuma, Ana Cuenca, Ralf Nauen, Celso Omoto

**Affiliations:** 1 Department of Entomology and Acarology, University of São Paulo (ESALQ/USP)-Piracicaba, São Paulo 13418-900, Brazil; daniela.okuma@bayer.com; 2 Bayer SA, Agronomic Solutions, Av. Dr. Roberto Moreira, 5005, EAE, Sao Paulo 13148-914, Brazil; ana.cuenca@bayer.com; 3 Bayer AG, Crop Science Division, R&D, Pest Control, Alfred Nobel Str. 50, 40789 Monheim am Rhein, Germany

**Keywords:** *Spodoptera frugiperda*, fall armyworm, corn, ryanodine receptor, chlorantraniliprole, flubendiamide, diamide insecticide, target-site resistance, I4790K mutation

## Abstract

**Simple Summary:**

Fall armyworm (FAW), *Spodoptera frugiperda*, is a destructive moth pest species on various crops, particularly corn. It is native to the tropical regions of the Western Hemisphere such as Brazil, but recently invaded Africa, India, China, and Australia. Its larval stages damage crops by feeding, and to keep them under damage thresholds, insecticide applications are common. Due to frequent insecticide applications, FAW evolved resistance to different chemical classes of insecticides, including diamides. Field relevant levels of diamide resistance are usually conferred by ryanodine receptor (RyR) mutations and compromising recommended label rates. Diamide resistance in FAW so far remained restricted to laboratory-selected strains. Here, we investigated the frequency of specific resistance mutations in field-collected Brazilian populations of FAW by an F2 screen, selected two populations (BA-R and TF-R) for high levels of diamide resistance, deciphered the genetics of resistance, and employed a molecular genotyping assay to correlate resistance levels with the presence of RyR mutations. Crossin studies indicated that resistance is autosomal and (incompletely) recessive in both strains. F1 backcrosses suggested monogenic resistance, supported by the identification of an I4734M/K target-site mutation in the RyR. Our results will help to sustainably manage diamide resistance in FAW in Brazil.

**Abstract:**

Fall armyworm (FAW), *Spodoptera frugiperda*, is an important lepidopteran pest in the Americas, and recently invaded the Eastern Hemisphere. In Brazil, FAW is considered the most destructive pest of corn and cotton. FAW has evolved resistance to many insecticides and *Bacillus thuringiensis* (Bt) proteins. Here, a large-scale monitoring was performed between 2019 and 2021 to assess diamide insecticide susceptibility in more than 65 FAW populations sampled in corn and cotton. We did not detect a significant shift in FAW susceptibility to flubendiamide, but a few populations were less affected by a discriminating rate. F2 screen results of 31 selected FAW populations across regions confirmed that the frequency of diamide resistance alleles remained rather stable. Two laboratory-selected strains exhibited high resistance ratios against flubendiamide, and cross-resistance to anthranilic diamides. Reciprocal crosses indicated that resistance is autosomal and (incompletely) recessive in both strains. F1 backcrosses suggested monogenic resistance, supported by the identification of an I4734M/K target-site mutation in the ryanodine receptor (RyR). Subsequent genotyping of field-collected samples employing a TaqMan-based allelic discrimination assay, revealed a low frequency of RyR I4790M/K mutations significantly correlated with phenotypic diamide resistance. Our findings will help to sustainably employ diamides in FAW resistance management strategies across crops.

## 1. Introduction

Fall armyworm (FAW), *Spodoptera frugiperda* (J.E. Smith) (Lepidoptera: Noctuidae), is a polyphagous pest responsible for great economic losses in many crops [1,2], especially row crops such as corn and cotton [3,4], but its occurrence and importance is also increasing in soybean fields in Brazil [5]. Native from the tropical Americas and globally recognized as one of the most important arthropod pests it recently invaded the Eastern Hemisphere where it was detected in western Africa in 2016 [6], from where it dispersed to sub-Saharan Africa [7], and in 2018, to Asia-Pacific, including India [8], China and Australia [9,10].

Chemical control by the application of insecticides and the use of transgenic plants expressing *Bacillus thuringiensis* (Bt) insecticidal proteins are the main tactics used for FAW control [11,12]. However, the dynamics of the cultivation of large crops in Brazil, combined with climatic conditions and the inappropriate and frequent use of these methods, contributed to the establishment of FAW resistance to various insecticides and Bt proteins currently commercialized. Cases of resistance in FAW have been reported for various chemical classes of insecticides such as pyrethroids [13], organophosphates and carbamates [14], benzoylureas [15], spinosyns [16], diamides [17,18] and avermectins [19], but also Bt insecticidal proteins such as Cry1Ab [20], Cry1F [21], Cry1Ac + Cry1F [22], Cry1A.105 + Cry2Ab2 [23] and Vip3Aa [24].

Diamide insecticides selectively act on insect ryanodine receptors (RyR), large tetrameric calcium release channels located in the sarcoplasmic reticulum in neuro-muscular tissue of insects [25,26]. Typical symptoms of poisoning are feeding cessation and uncontrolled muscle contractions eventually leading to death. Diamides are nowadays an important tool of integrated pest management (IPM) due their high efficacy and selectivity [27,28]. Until nowadays, they have been among the most important tools for lepidopteran pest control in soybean, corn and cotton corresponding to more than 20% of the market share in Brazil in 2021 [29]. Flubendiamide, a phthalic acid diamide, was introduced to the market in 2007 [28], followed by the anthranilic diamides chlorantraniliprole and cyantraniliprole [30,31,32]. Both chemotypes activate insect RyRs by binding to a common site in the C-terminal transmembrane domain of the RyR [33,34,35], leading to the uncontrolled release of calcium from intracellular stores.

Diamide insecticide resistance was first reported in *Plutella xylostella* [36,37] followed by at least nine other lepidopteran species (for a review, see [38]), including FAW [18]. However, diamide resistance in field-collected populations of FAW compromising recommended label rates has not been reported yet, despite high levels of FAW resistance having been detected in Puerto Rican field strains [39]. Much higher levels of resistance, indeed, compromising label rates of chlorantraniliprole and flubendiamide, have been described in a laboratory-selected Brazilian strain [18]. Resistance to diamides is mainly conferred by RyR target-site mutations such as G4946E and I4790M (*P. xylostella* RyR numbering) and were first reported in diamondback moths collected in the Philippines, Thailand and China [36,40], followed by populations collected in other geographies including India, Taiwan, USA, Japan and Korea [41]. Later, similar mutations at RyR positions I4790 and G4946 have been described in *Tuta absoluta* [42], *Chilo suppressalis* [43,44] and *Spodoptera exigua* [45,46]. Mutations at RyR positions I4790 and G4946 and their impact on diamide binding and resistance have been functionally validated, e.g., by radioligand binding studies [41], recombinant expression of mutant receptors in insects cell lines [47,48] and by CRISPR/Cas9 genome-edited transgenic *Drosophila* [49] and target species such as *Spodoptera exigua* [50].

The development of target-site resistance to diamides is a main threat to the sustainability of the entire chemical group. The agriculture practice in Brazil is typically based on two or more crop seasons per year, allowing pest populations to go through multiple generations per year and consequently increasing selection pressure to insecticides. Resistance management strategies for diamides have been reported but are often not consequently implemented [51]. Therefore, the understanding of the resistance status to a target pest such as FAW at the field level can contribute to resistance risk assessment for existing and novel diamides.

In this study, we carried out a three-year large-scale assessment on commercial corn and cotton fields in Brazil after ten years of flubendiamide commercial launch with the objectives (a) to evaluate flubendiamide susceptibility in more than 65 field-collected FAW populations collected between 2019–2021, (b) to assess the resistance allele frequency by genotyping and mapping potential RyR mutations utilizing an F2 screen, (c) to establish resistant strains by selection to investigate their cross-resistance to other diamides and the genetics of resistance, and (d) to understand how specific RyR target-site mutations contribute to the diamide resistance.

## 2. Materials and Methods

### 2.1. Fall Armyworm (FAW) Strains

Field populations of *Spodoptera frugiperda*, fall armyworm (FAW), were collected as larvae in Brazilian maize and cotton fields over three consecutive seasons (Table S1; Figure S1). They were maintained as described below and tested once they reached F1. FAW strains resistant to flubendiamide, BA-R and TF-R were selected from field populations of *S. frugiperda* collected in São Desidério (13°15′51.70″ S, 46°08′45.82″ E) (BA-R) during season 2017/2018 and Tasso Fragoso (7°49′33.8″ S, 46°00′22.6″ E) (TF-R) in 2018/2019, using the F2 screen methodology as described below [52]. For the resistant strain selection, 128 third instar larvae were tested from 63 isolines for BA-R, and from 91 isolines for TF-R. The larvae were exposed to flubendiamide (Belt^®^, Bayer S/A, Sao Paulo, Brazil) (480 g/L) in diet overlay bioassay using the discriminating dose as described below. The survivors were selected, raised on an artificial diet and maintained with continuous selection pressure of 22.7 μg ai/cm^2^ of flubendiamide. Isolines with surviving larvae were considered positive and used to estimate the resistance allele frequency in field populations. Based on the algorithm described earlier [53], resistance allele frequency was estimated using the function binom.bayes from the package binom in R statistical software [54]. The susceptible strain (Sus) has been maintained in laboratory for more than 10 years, free of selection pressure by insecticides and Bt proteins. FAW were maintained on artificial diet under controlled conditions (25 ± 1 °C, 70 ± 10% relative humidity, and a 14:10 h light/dark photoperiod) during all phases of development as described earlier [18].

### 2.2. Phenotypic Resistance Monitoring of Faw Using a Diagnostic Concentration of Flubendiamide

Diet overlay bioassays were performed with flubendiamide (Belt^®^) (480 g a.i./L) using a diagnostic concentration of 0.41 µg a.i./cm^2^ (LC_99_) based on a previous study with FAW [55]. The bioassays were performed in a 128-well bioassay tray (BIO-BA-128; CD International Inc., Pitman, NJ, USA), containing 1 mL of diet in each well. For the bioassays, formulated flubendiamide (Belt^®^ 480 g a.i./L) was diluted in 0.1% aqueous Triton X-100. Thirty microliters were applied onto the diet surface in each well. After drying, one third instar larva from F1 generation was placed into each well. Trays were sealed with a plastic adhesive (BIO-CV-16; CD International Inc., NJ, USA) that allowed air exchange and kept in a climatic chamber at 25° ± 2 °C, 60% ± 10% RH and 14:10 h (L:D) photoperiod. Mortality was recorded after 4 days. A total of 512 larva/population were tested and 67 populations were sampled in the seasons 2018/19 to 2020/21 (Table S1, Figure S1).

### 2.3. F2 Screen for Flubendiamide Resistance Alleles

To estimate flubendiamide resistance allele frequency in Brazilian populations of FAW, we utilized the F2 screen method [52]. From 2018/19 (2019) to 2019/20 (2020), a total of 31 populations of FAW were screened (Table S2). Field collected larvae were transported to the laboratory and kept on artificial diet until pupation. The respective pupae were separated by sex and used to establish multiple single-pair mating couples under laboratory conditions. The offspring (F1 progeny) of each single-pair mating (isoline) were reared on artificial diet, and pupae were transferred to a cylindrical PVC cage (20 cm height × 10 cm diameter) lined with paper (oviposition substrate) and covered with a mesh fabric until adult emergence. Adults were fed with a 10% honey solution provided on cotton inside a plastic cup. Eggs were collected and kept in plastic cups with filter paper moistened with water until larval eclosion. F2 generation third instar larvae were then used for screening. For the bioassays, a discriminating flubendiamide concentration of 12.71 µg a.i./cm^2^ was used. Each isoline was tested in 128-well bioassay trays (BIO-BA-128; CD International Inc., NJ, USA) containing 1 mL of artificial diet following the same methodology as described above. One third instar larva was placed in each well, with a target number of 128 neonates tested/isoline. In total, 1044 isolines were tested in 2018/19 (2019), 461 isolines in 2019/20 (2020) and 469 isolines in 2020/21 (2021). Then, plates were sealed and placed under the same environmental conditions described above. Survivorship was recorded after 4 days. An isoline was considered positive (putatively resistant) if it reached the adult stage. To estimate the resistance allele frequency, we used the equations published earlier [52,53]. The resistance allele frequency was calculated using the function binom.bayes from the package binom in R statistical software—R v.4.2.0 [54].

### 2.4. Genetics and Inheritance of S. frugiperda Resistance to Flubendiamide

To characterize the genetics of resistance, we used the same bioassay as described above and tested eight to eleven concentrations of flubendiamide (Belt^®^) (480 g/L). As a control treatment, aqueous 0.1% Triton X100 was used. At least four replicates with 16 larvae each were tested for each treatment. Lethal concentrations (LC_50_) and 95% confidence intervals were estimated using probit analysis in R statistical software [54]. The resistance ratio (RR) was estimated by dividing the LC_50_ of the resistant strain by the LC_50_ of Sus strain.

#### 2.4.1. Dominance of Resistance

Reciprocal crosses were performed to investigate the inheritance pattern of flubendiamide resistance in two selected strains, BA-R and TF-R employing the same method as previously described [18]. Thirty pairs of each heterozygous strain H1 (BA-R/TF-R ♂ × Sus ♀) and H2 (BA-R/TF-R ♀ × Sus ♂) were transferred to PVC cages to allow mating and oviposition. Third instar larvae progeny from the heterozygous strains were exposed to concentration-response bioassays, as described above. The degree of dominance (D) for each concentration tested was calculated as described earlier [56,57].

#### 2.4.2. Number of Genes Associated with Resistance

The number of genes associated with flubendiamide resistance in FAW was estimated using procedures described earlier [58,59]. Briefly: F1 progeny from the heterozygous strains H1 and H2 (see above) were backcrossed with individuals from the resistant strains (BA-R and TF-R, separately). Thirty pairs were formed for each backcross, i.e., BA-R/TF ♂ × F1 ♀ and BA-R/TF ♀ × F1 ♂, and the resulting 3^rd^ instar larval progeny was tested as described above. The hypothesis of monogenic inheritance was tested using the chi-square (χ²) test and the number of loci associated with resistance was estimated using the method described by Lande [60].

### 2.5. Cross-Resistance of Flubendiamide Resistance to Anthranilic Diamides

Cross-resistance to chlorantraniliprole (Premio^®^, FMC, USA (200 g a.i./L) and cyantraniliprole (Benevia^®^, FMC, USA (200 g a.i./L) of FAW strains BA-R and TF-R in comparison to strain Sus was investigated using the same diet overlay bioassay as described above. Dose response data were subjected to probit analysis utilizing R statistical software [54]. Parallelism and equality tests (*p* < 0.05) were also performed to compare the angular and linear coefficients of the respective probit line obtained for each strain [61].

### 2.6. Partial Sequencing of the Ryanodine Receptor (RyR) C-Terminal Transmembrane Domain

Total RNA was extracted from 10 third instar larvae of FAW strain Sus, BA-R and TF-R using an RNeasy Mini Kit^®^ extraction kit (Qiagen, Hilden, Germany), according to the manufacturer’s specifications. The quality and concentration of the isolated RNA were verified by spectrophotometry (NanoDrop^®^ One, ThermoFisher Scientific, Waltham, MA, USA). The synthesis of cDNA was performed using 2 μg of total RNA treated with DNAse. For the reverse transcriptase reaction, the commercial system GoScript ™ Reverse Transcriptase (Promega, Madison, WI, USA) was used, according to the manufacturer instructions.

The primer pairs used, i.e., 1-F, 1-R, 2-F, 3-F and 2-R to amplify overlapping RyR fragments were exactly the same as described previously [17] (Table S3). PCR amplification was performed in a 25 µL reaction mix containing 2.5 µL 10× reaction Buffer (Promega, Madison, WI, USA), 0.5 µL 10mM dNTP, 0.2 µL 200 µM of each primer, 60 ng cDNA, 0.5 units Pfu DNA Polymerase (Promega, Madison, WI, USA). The amplification reactions were conducted at 98 °C (10 s), followed by 30 cycles of 98 °C (1 s), 50 °C/52 °C/60 °C (1F + 1R/2F + 1R/3F + 2R) (5 s) and a final extension step at 72 °C for 2 min using a ViiATM7 Real-Time PCR (Applied Biosystems, Foster City, CA, USA). The efficiency of the amplification reaction was verified via electrophoresis in 1.5% agarose gel (7 µL ethidium bromide, in a tris-acetate-EDTA (TAE) buffer solution (40 mM Tris-acetate, 1 mM EDTA, pH 7.2) at a constant voltage of 100 V and subsequent visualization in a transilluminator. The samples were purified with ExoSAP-IT™ (Applied Biosystems™, Waltham, MA, USA) and custom Sanger sequenced by The Central Laboratory of High-Performance Technologies (LCTAD, Campinas, Brazil). The obtained sequences were submitted to GenBank and the following accession numbers were assigned: Sf_Sus (MK805909.1), BA-R (ON653045), TF-R 4790M (ON653048), TF-R 4790K (ON653047) and TF-R 4790K/M (ON653046). The obtained sequences were aligned with the corresponding sequences of *S. exigua* (GenBank KJ573633), *P. xylostella* (GenBank AET09964) *T. absoluta* (GenBank APC65631) and *S. frugiperda* Sus (GenBank MK805909.1) using Geneious software v. 10.2.3 (Biomatters Ltd., Auckland, New Zealand).

### 2.7. PCR-Based Allelic Discrimination Assay for Genotyping

Probes containing different fluorescent dyes were used for single allele detection of wildtype and mutant gene fragments in a modified real-time PCR assay. Primers and probes (Table S3) for the detection of I4790 and M4790 alleles (*P. xylostella* RyR numbering) were based on Boaventura et al. [17], whereas probes for K4790 detection were designed using Geneious v.10.2.3 (Biomatters Ltd., Auckland, New Zealand). Individuals with a known genotype from FAW strain Sus, resistant strains BA-R and TF-R and heterozygotes were tested and PCR was conducted using a ViiATM7 Real-Time PCR (Applied Biosystems, Foster City, CA, USA) with end-point fluorescence values taken at cycle 35. On average, twenty moths from 64 field populations collected during crops seasons 2018/2019, 2019/2020, 2020/2021 were used to map the frequency of different alleles (Table S4). Resistance allele frequency detected in the PCR-based allelic discrimination assay was correlated with the survivorship observed in the phenotypic flubendiamide discriminating dose monitoring. Analyses were performed in GraphPad Prism 9 (GraphPad Software, San Diego, CA, USA).

## 3. Results

### 3.1. Phenotypic Resistance Monitoring of FAW Using a Diagnostic Concentration of Flubendiamide

**Figure 1:**
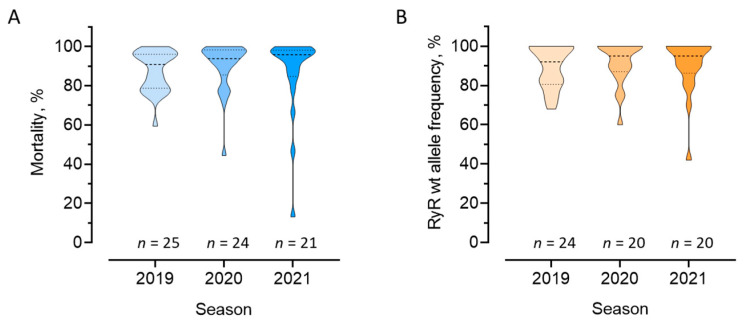
Phenotyping and genotyping of diamide resistance in Brazilian Spodoptera frugiperda sampled across three seasons. Violin plots of the efficacy of a discriminating rate of flubendiamide (diet-overlay assay, 0.41 μg a.i./cm^2^) against 70 field populations of fall armyworm (**A**), and ryanodine receptor (RyR) I4790 wildtype (wt) allele frequency in 64 fall armyworm populations (**B**), sampled across three cropping seasons (2019–2021). One-way ANOVA and Tukey’s post-hoc multiple comparisons test revealed no significant differences between seasons (*p* > 0.05).

Most of the field populations collected during the cropping seasons 2019, 2020 and 2021 showed rather high mortality >90% when exposed to a flubendiamide diagnostic concentration of 0.41 μg a.i./cm^2^ (Figure 1A). Third instar larval mortality of populations collected in 2019, 2020 and 2021 ranged from 75% to 100%, 71% to 100%, 46.7% to 100%, respectively, with FAW populations sampled in Bahia showing the lowest mortality (Figure S2). Our results revealed no significant shift in flubendiamide susceptibility between 2019 and 2021 (*p* > 0.05, one-way ANOVA, followed by Tukey’s post-hoc multiple comparisons test). However, FAW populations collected in Bahia state showed consistently lower susceptibility to flubendiamide in all cropping seasons, varying from 59.4% in 2019 to 46.7% in 2021, and even 13.2% mortality in a single FAW population collected off-season in 2021 in Sao Desidério on cotton (Figure S2).

### 3.2. Assessment of Flubendiamide Resistance Allele Frequency

**Figure 2:**
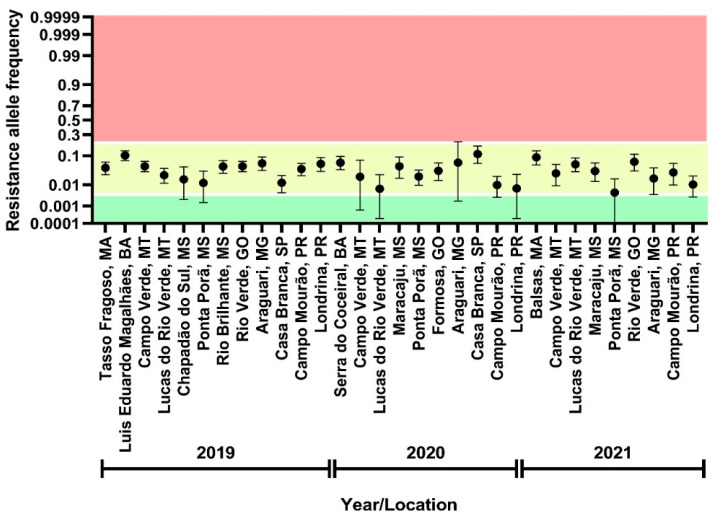
F2 screen results. Frequency of resistance alleles conferring resistance to flubendiamide in Brazilian populations of *Spodoptera frugiperda* employing F2 screen methodology based on sib-mated F1 isolines of field-collected female adults. In total, 1044, 461 and 469 isolines were tested in 2019, 2020 and 2021, respectively.

Out of all FAW populations collected between 2019 and 2021, a total of 31 populations, 1974 isolines and more than a hundred thousand third instar larvae were screened using a discriminating rate of flubendiamide (Table S4). Resistance alleles were detected across all populations in 295 isolines which were considered positive when reaching the adult stage under selection pressure. The obtained data for 31 FAW populations F2 screened are summarized in Figure 2 and Table S2. The estimated composite resistance allele frequency between seasons 2019 and 2020 was significantly different based on non-overlapping 95% confidence intervals (CI 95%): 2019, 0.0423 (CI 95%, 0.0364–0.0486) and 2020, 0.0275 (CI 95%, 0.0205–0.0354). However, the estimated mean resistance allele frequency in FAW populations collected in 2021 (0.0375 (CI 95%, 0.0272–0.0435) was not significantly different from mean values obtained for 2019 and 2020. Thus, suggesting no relevant shift in flubendiamide susceptibility and a rather stable resistance allele frequency across seasons (Figure 2), ranging between 0.0046 to 0.112 (Table S2).

### 3.3. Establishment of Flubendiamide Resistant FAW Strains BA-R and TF-R

Out of the 63 isolines screened from São Desidério (Bahia State) using the F2 screen methodology, 13 isolines were positive (BA-R), i.e., reaching adulthood at the discriminating rate of flubendiamide, and showing an allele frequency of 5.56% (CI 95%, 3.08–8.7). Out of the 91 isolines from Tasso Fragoso (Maranao State), 16 isolines were positive (TF-R) corresponding to a resistance allele frequency of 0.2% (CI 95%, 0.01–0.86). The laboratory-selected strains BA-R and TF-R exhibited high levels of flubendiamide resistance (LC_50_-values > 227 µg/cm²) with resistance ratios (RR) of > 4464-fold when compared to the Sus strain (Table 1).

### 3.4. Genetics of Resistance in FAW Strains BA-R and TF-R

The concentration–response bioassays conducted with progeny (F1) from the reciprocal crosses of strains BA-R and TF-R with strain Sus showed similar LC_50_ values with overlapping confidence intervals (Table 1). The resistance ratio for reciprocal crosses ranged from 2.12- to 2.98-fold for BA-R and 1.41- to 1.55-fold for TF-R, indicating an autosomal inherited trait (Table 1). The level of dominance according to Stone [57] revealed values < −0.74 for all crossings (values of −1 are completely recessive and +1 completely dominant), suggesting an almost recessive mode of inheritance for both selected strains. This is supported by dominance levels calculated according to Bourguet [56] and based on their decrease as a function of increasing concentrations of flubendiamide (Table 2). At concentrations close to the flubendiamide field-rate (72 g/ha; ~0.72 µg a.i./cm^2^), the dominance was less than 0.18 for BA-R and less than 0.10 for TF-R indicating an incomplete recessive mode of inheritance.

Observed and expected mortality values analyzed by Chi-square test (χ^2^) were significantly different (*p* < 0.05) only at the lowest concentration tested for strain BA-R (0.02 µg a.i./cm^2^). No significant difference at all was observed among the concentrations tested for strain TF-R, suggesting that flubendiamide resistance is conferred by a monogenic trait (Table 3). The number of calculated independent segregations was 1.42 to 2.84 for BA-R and 1.3 to 1.77 for TF-R, suggesting that the number of loci associated with flubendiamide resistance in *S. frugiperda* is low.

### 3.5. Cross-Resistance of FAW Strains BA-R and TF-R to Anthranilic DIamides

The flubendiamide resistant strains BA-R and TF-R exhibited strong cross-resistance against the anthranilic diamides, chlorantraniliprole and cyantraniliprole. Depending on the strain and diamide tested, resistance ratios between 713-fold and 6400-fold were obtained when compared to the Sus strain (Table 4). Significant differences in susceptibility to anthranilic diamides between both strains were confirmed by equality (chlorantraniliprole: χ^2^ = 230.0; df = 2; *p* < 0.001; cyantraniliprole: χ^2^ = 230.0; df = 2; *p* < 0.001) and parallelism (chlorantraniliprole: χ^2^ = 130.3; df = 1; *p* < 0.001, cyantraniliprole: χ^2^ = 230.0; df = 2; *p* < 0.001) tests.

### 3.6. Ryanodine Receptor (RyR) C-Terminal Transmembrane Sequencing

**Figure 3:**
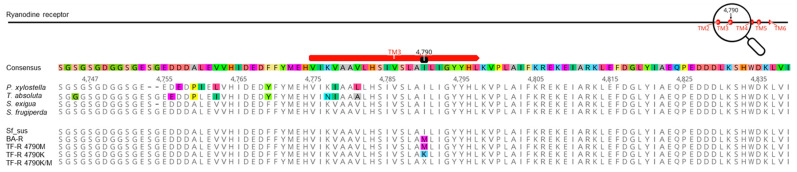
Ryanodine receptor (RyR) partial sequence alignment. Multiple alignment of the amino acid sequence of a partial region of the RyR of different lepidopteran species comprising the I4790M/K region linked to diamide resistance in selected Brazilian strains BA-R and TF-R of *Spodoptera frugiperda*. The accession numbers of the shown sequences are: *Plutella xylostella* (AET09964), *Tuta absoluta* (APC65631), *Spodoptera exigua* (AFC36359), *S. frugiperda* (MK226188), Sf_Sus (MK805909.1), BA-R (ON653045), TF-R 4790M (ON653048), TF-R 4790K (ON653047) and TF-R 4790K/M (ON653046).

We amplified and sequenced a 1410 bp partial transmembrane fragment (domains II-VI) of the RyR of strains Sus, BA-R and TF-R encompassing sites I4790 and G4946 (*P. xylostella* RyR numbering), and the obtained fragments showed high similarity at the amino acid level and revealed only non-synonymous SNPs causing either an I4790M mutation (strain BA-R and TF-R) and/or an I4790K mutation (strain TF-R) (Figure 3; see also for GenBank accession numbers). All individuals sequenced by Sanger sequencing were either homozygote for the wild-type allele I4790 (strain Sus), homozygous M4790 (BA-R 100%, TF-R 40%), homozygous K4790 (TF-R 40%) or heterozygous M/K4790 (TF-R 20%) —only detected in individuals of strain TF-R (Figure S3).

### 3.7. Genotyping of FAW Field Samples by a PCR-Based Allelic Discrimination Assay

**Figure 4:**
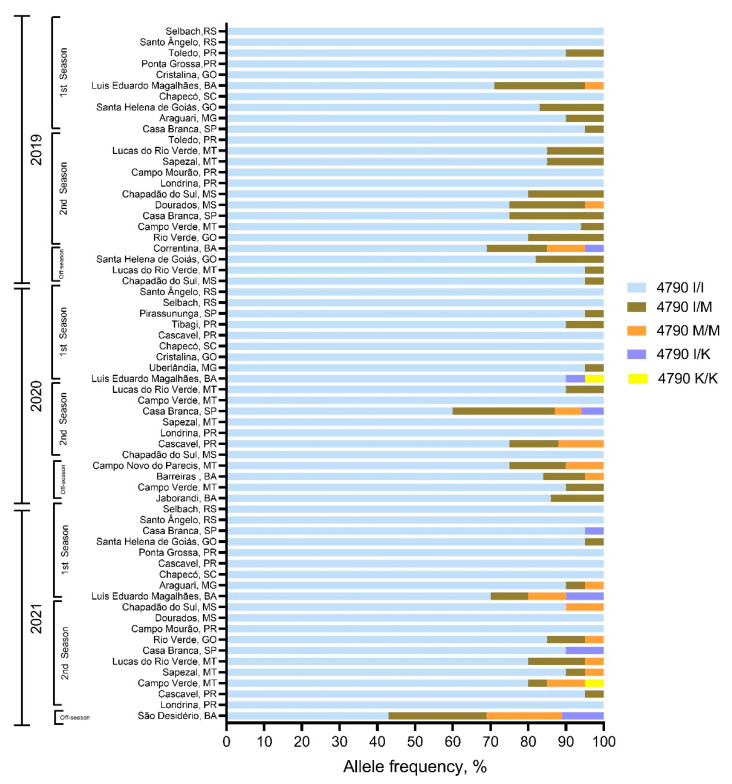
Genotyping of diamide resistance allele frequency in Brazilian populations of *Spodoptera frugiperda*. Homozygous and heterozygous ryanodine receptor (RyR) allele frequencies in field-collected fall armyworm samples. Samples were analyzed employing a PCR-based allelic discrimination assay for the presence of RyR resistance alleles I4790 (wildtype), M4790 and K4790 (resistant) (numbering according to *Plutella xylostella* RyR, GenBank JN801028). For a detailed data table depicting the number of analyzed individuals per location, please refer to the Supplementary Materials (Table S4).

In total, more than 1000 individuals from 64 FAW populations out of those collected in the seasons 2018/2019 to 2020/2021 have been analyzed using the developed TaqMan assay. By far, the largest portion was wildtype I4790 (Figure 1B), as expected, considering the large-scale monitoring data and the outcome of the F2 screen. However, 39 FAW field-populations tested positive for the presence of resistance alleles (M4790, K4790) in at least one analyzed individual (Figure 4).

None of the populations collected in Rio Grande do Sul (RS) in the south of Brazil was tested positive for the presence of any resistance allele, whereas RyR mutations were detected in most FAW populations from Mato Grosso (MT), Sao Paulo (SP) and Bahia (BA) state (Figure 4, Table S4). A higher frequency of resistance alleles was detected in FAW from MT and BA state, correlating with the phenotyping results obtained for these populations. Particularly FAW samples collected after the second season of corn showed a higher frequency of resistance alleles (Figure 4), possibly indicating survivors of diamide selection pressure applied in the first season. Most of the detected genotypes are either heterozygous I4790/M4790 or homozygous M4790. The prevalent M4790K substitution detected in the selected strain TF-R was very rarely detected among the field-collected populations (<10% out of those populations tested positive). Only two individuals (one from MT, and one from BA) were homozygote K4790, suggesting at present a minor role of this resistance allele. The ranking of the detected RyR genotypes based on abundance across FAW samples and seasons is as follows: I4790 >>> I/M4790 >> M4790 >> I/K4790 > K/K4790.

**Figure 5:**
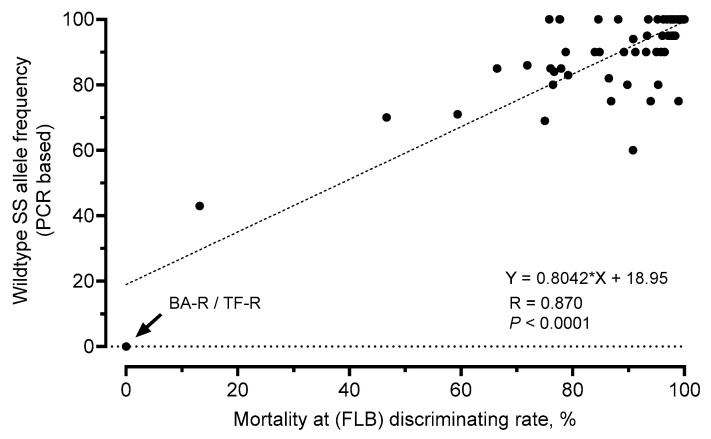
Relationship between diamide efficacy and resistance allele frequency in *Spodoptera frugiperda*. Linear regression analysis revealed a significant correlation (F = 188, df = 1, 60, *p* < 0.0001) between ryanodine receptor resistance allele frequency (M4790 and K4790) and survival of field-collected fall armyworm populations at a discriminating rate of flubendiamide (FLB; diet-overlay assay, 0.41 μg a.i./cm^2^).

A regression analysis revealed that the obtained mortality rates of the large-scale phenotypic screening employing a discriminating rate of flubendiamide were significantly correlated with a decreasing abundance of wildtype RyR I4790 alleles as determined by the PCR-based allelic discrimination assay (F = 188.0, df = 1, 60, R = 0.870, *p* < 0.0001) (Figure 5). It needs to be mentioned that this analysis largely ignores susceptibility issues caused by genotypic variations in FAW populations, likely to be conferred by a suit of different mutations. We showed, for example, in crossing experiments with strains BA-R and TF-R that heterozygous genotypes express an almost susceptible phenotype. However, excluding strains BA-R and TF-R still results in a significant correlation between genotyping and phenotyping data (F = 54.32, df = 1, 58, R = 0.695, *p* < 0.0001).

## 4. Discussion

In general, the phenotypic monitoring of flubendiamide resistance did not confirm shifts in the susceptibility of FAW between 2018–2021 that would lead to unexpected field control failures. An initial shift was observed in resistance monitoring initiatives carried out in Brazil during the cropping seasons 2011/12 to 2013/14 [55] and 2015/16 to 2017/18 [18], mainly in cotton producer regions (MT and BA) and maize seed-production areas (Casa Branca, SP). However, our study indicated that the diamide resistance allele frequency was rather stable and did not significantly increase between seasons 2018/2019 and 2020/2021.

To further characterize the potential flubendiamide resistance alleles detected during the F2 screen, we have chosen two different populations, BA-R and TF-R, which were further selected and showing high levels of flubendiamide resistance (RR > 4700-fold) compromising the efficacy of recommended field rates. Other studies reported 10-fold higher resistance levels to flubendiamide resulting from chlorantraniliprole laboratory-selection of a field-collected Brazilian FAW population [18]. However, diamide resistance ratios in FAW selected under applied conditions have been much lower and unlikely to result in complete control failure [39]. In contrast, field-collected populations of other destructive lepidopteran pest species have been reported to exhibit high levels of diamide resistance resulting in control failure, e.g., diamondback moth [36,37,41,48], beet armyworm [45,62], tomato leafminer [42,63] and smaller tea tortrix [64].

The flubendiamide selected strains BA-R and TF-R showed high levels of cross-resistance to the anthranilic diamides chlorantraniliprole and cyantraniliprole. Similar cross-resistance issues have been also reported in other species such as *Plutella xylostella* and *Spodoptera exigua* [41,45,65], and are therefore not unexpected, as flubendiamide and anthranilic diamides represent a single chemical class [27], sharing the same mode of action within the Insecticide Resistance Action Committee (IRAC) mode of action classification scheme [66]. As expected from F2 screen bioassay data and reciprocal (back)crossings, the inheritance of flubendiamide resistance in strains BA-R and TF-R followed an autosomal, monogenic incompletely recessive pattern. This is in line with previous genetic studies on diamide resistance in FAW and other lepidopteran pests, and consistent with the reported presence of RyR target-site mutations in the C-terminal domain [18,41,42,67].

The mechanisms of diamide resistance so far identified are almost exclusively due to target-site mutations in the region II to VI of the RyR transmembrane domain, reviewed in [38]. In this study, we detected the presence of two different amino acid substitutions in FAW strains BA-R and TF-R at the RyR position I4790 (*P. xylostella* RyR numbering) and associated with diamide resistance. First, I4790M, formerly described in *P. xylostella* [40,65], *Tuta absoluta* [42], *S. exigua* [46] and *S. frugiperda* [17], and second, I4790K, only recently described for the first time in *P. xylostella* [65,68]. I4790K mutation is of particular interest as it has been shown to result in high diamide resistance levels in *P. xylostella* across chemotypes, whereas the impact of I4790M was much lower [68]. The profound impact of I4790K on diamide binding to RyR has been functionally validated by electrophysiological means employing an I4790K engineered *Bombyx mori* RyR recombinantly expressed in HEK293 cells [65]. The importance of I4790M for diamide resistance has been functionally validated by different approaches, such as in vivo introgression of the I4790M mutation into a wildtype *S. exigua* line resulting in 20-fold resistance to both chlorantraniliprole and flubendiamide [46]. Another study integrated the entire coding sequence of a mutated *P. xylostella* RyR into *Drosophila melanogaster*, where the I4790M variant conferred 22-fold resistance to flubendiamide but only 4-fold resistance to chlorantraniliprole, thus confirming its lower impact on resistance to diamide insecticides [47]. A similar pattern was also described for a chlorantraniliprole-selected FAW strain homozygous for I4790M, and showing much higher resistance to flubendiamide (>5400-fold) than to chlorantraniliprole (225-fold) [17]. It has been shown by *P. xylostella* RyR homology modeling in complex with chlorantraniliprole-based on a rabbit RyR1 cryo-EM structure-that I4790 is one of the insect-specific residues involved in diamide binding [34]. Its role as one of the key amino acids into the diamide binding site was recently also confirmed in chimeric *P. xylostella* RyR constructs, where isoleucine at position 4790 was replaced by a cysteine found in the diamide insensitive skeletal RyR1 of humans. It has been shown that I4790C-mutated *P. xylostella* RyR functionally expressed in Sf9-cells showed strongly reduce diamide efficacy (detected via calcium imaging) at low-to mid-nanomolar concentrations [69]. Functional studies with recombinantly expressed I4790M/K mutated FAW RyRs are lacking as yet, but would help to support the in vivo results obtained here for strains BA-R and TF-R.

Employing a PCR-based allelic discrimination assay, we detected for the first time the co-occurrence of the I4790M and I4790K mutations in several FAW field populations in Brazil. Similar PCR-based investigations tests have been conducted in the past but failed to detect such mutations in FAW field populations collected across different continents [70]. Two other studies with diverse field-collected FAW populations recruiting whole genome sequencing approaches also failed to detect RyR mutations at sites I4790 and G4946 [71,72].

Our genotyping assays revealed that the I4790M mutation is present in FAW populations across several Brazilian states, except for Rio Grande do Sul state, where winter temperatures allow just one corn planting season. In contrast, I4790K mutation is present in a few regions only with higher selection pressure in the states of Bahia, Mato Grosso and São Paulo, specifically, in cotton and corn seed-production areas with two or more cropping seasons per year. Our F2 screen results and the low abundance of the I4790K mutation suggest a rather low risk of diamide resistance evolution in FAW. This is corroborated by high fitness costs associated with diamide resistance in FAW, a major factor influencing the rapid evolution of resistance to diamides in Brazil [73], especially if the higher fitness cost is associated with the mutation I4790M in resistant field-evolved populations. In addition, the significant reduction in the number of insecticide sprays to manage lepidopteran larvae over the seasons and crops, and the increase in adoption of insecticides from different modes of actions apparently delayed the resistance evolution of FAW to diamides (Figure S4).

To maintain the benefits and efficacy of diamide-based products in FAW control it is extremely important to follow integrated pest management strategies and to adopt them as needed at local level [74]. Diamide insecticides will continue to be an important tool for global pest management tactics; therefore, the implementation of resistance management strategies is essential for the sustainability of existing and new products [51].

## 5. Conclusions

In conclusion, we confirmed the results of previous studies that diamide resistance in FAW is an incomplete recessive trait, costly, and that it is unlikely to increase in case sustainable resistance management strategies are implemented. Our work reinforces the importance of the knowledge of the genetics and mechanisms of resistance and to continuously monitor the frequency of resistance alleles in field populations as a basis for a robust resistance management program.

## Figures and Tables

**Table 1:** Log-dose probit-mortality data for flubendiamide tested against larvae of resistant (BA-R and TF-R), susceptible (Sus) and reciprocal F1 crosses of *Spodoptera frugiperda*. The degree of dominance (D) was calculated according to Stone [57].

Strain	N	LC_50_ (95% CI) ^a^ [μg a.i./cm^2^]	Slope ± SE	χ²	Df ^b^	RR ^c^	D
Sus	768	0.05 (0.04–0.07)	1.53 ± 0.11	16.2	9	-	-
BA-R	784	>227	0.70 ± 0.26	2.8	10	>4464	-
Sus♀ × BA-R♂	832	0.11 (0.07–0.15)	1.38 ± 0.12	18.1	9	2.12	<−0.82
Sus♂ × BA-R♀	832	0.15 (0.10–0.22)	1.38 ± 0.13	26.3	10	2.98	<−0.74
TF-R	512	>227	0.80 ± 0.13	3.4	5	>4464	-
Sus♀ × TF-R♂	512	0.07 (0.05–0.10)	1.53 ± 0.13	9.73	7	1.41	<−0.92
Sus♂ × TF-R♀	496	0.08 (0.05–0.11)	1.29 ± 0.11	8.67	7	1.55	<−0.89

^a^ Confidence interval, 95%; ^b^ degrees of freedom; ^c^ Resistance ratio (LC_50_ of tested strain divided by LC_50_ of Sus).

**Table 2:** Degree of dominance (D) according to Bourget [56] in reciprocal crossings of different strains of *Spodoptera frugiperda* as a function of increasing flubendiamide concentration.

Flubendiamide (μg a.i./cm^2^)	Sus	BA-R ♂ × Sus ♀		TF-R ♂ × Sus ♀		BA-R	TF-R
	Mortality (%)	Mortality (%)	Dominance (D)	Mortality (%)	Dominance (D)	Mortality (%)	Mortality (%)
0.00	2.08	0.00	1.00	0.00	1.00	0.00	0.00
0.01	14.58	3.13	0.79	21.87	0.50	0.00	0.00
0.02	36.46	4.69	0.87	25.00	0.31	0.00	0.00
0.13	64.58	46.88	0.27	59.38	0.08	0.00	0.00
0.41	87.50	71.35	0.18	78.15	0.10	0.00	0.00
1.27	100.00	86.98	0.13	97.92	0.02	0.00	0.00
2.27	100.00	92.19	0.08	100.00	0.00	0.00	0.00
22.70	100.00	98.44	0.02	100.00	0.00	2.08	4.69
227.00	100.00	100.00	0.00	100.00	0.00	6.25	29.69

**Table 3:** Chi-square analysis (χ^2^) of the mortality data from the backcrosses between *S. frugiperda* resistant to flubendiamide (BA-R and TF-R) and F1 progeny of the reciprocal crosses exposed to different concentrations of flubendiamide. Obs, observed; Exp, expected, based on Mendelian inheritance. * Indicates a significant difference (*p* < 0.05) between observed and expected mortalities.

Flubendiamide (μg a.i./cm^2^)	BA-R						TF-R					
	BA-R ♀ x F_1_ ♂			BA-R ♂ x F_1_ ♀			TF-R ♀ x F_1_ ♂			TF-R ♂ x F_1_ ♀		
	Obs.	Exp.	χ^2^	Obs.	Exp.	χ^2^	Obs.	Exp.	χ^2^	Obs.	Exp.	χ^2^
0.02	12.5	2.34	28.84 *	10.42	2.34	18.22 *	0	19.53	0.04	0	19.53	0.04
0.23	29.69	28.9	0.02	36.88	28.9	1.98	17.97	37.5	0.30	6.25	37.5	0.10
2.27	31.25	39.06	1.64	43.75	39.06	0.59	45.32	50	0.45	40.18	50	0.40
12.71	39.58	47.92	1.78	46.88	47.92	0.03	51.04	50	0.51	43.75	50	0.44
22.70	45.83	49.71	0.38	43.75	49.71	0.91	48.22	52.1	0.44	48.44	52.1	0.44
227.00	59.38	50.5	2.02	53.13	50.5	0.18	70	64.58	0.38	69.79	64.58	0.38

**Table 4:** Log-dose probit-mortality data for anthranilic diamide insecticides tested against larvae of different strains of *Spodoptera frugiperda*.

Compound	Strain	n	LC_50_ (95% CI) ^a^ (µg a.i./cm²)	Slope ± SE	χ²	Df ^b^	RR ^c^
Chlorantraniliprole	Sus	464	0.010 (0.007–0.014)	1.33 ± 0.10	5.25	10	-
	BA-R	496	7.13 (4.33–13.0)	1.73 ± 0.08	18.2	8	713
	TF-R	448	40.4 (27.0–55.3)	1.02 ± 0.08	10.6	7	4040
Cyantraniliprole	Sus	224	0.002 (0.001–0.003)	3.64 ± 0.66	0.74	6	-
	BA-R	384	3.09 (0.59–6.29)	1.62 ± 0.16	21.3	6	1545
	TF-R	800	12.8 (6.87–21.2)	1.13 ± 0.07	12.8	7	6400

^a^ Confidence interval, 95%; ^b^ degrees of freedom; ^c^ Resistance ratio (LC_50_ of strain BA-R/TF-R divided by the LC_50_ of strain Sus).

## Data Availability

The data supporting the conclusions of this article are provided within the article. The original datasets analyzed in this study are available from the corresponding author upon request. The nucleotide sequences reported in this study have been deposited in the GenBank database under the accession numbers ON653045, ON653048, ON653047 and ON653046.

## Supplementary Materials

The following supporting information can be downloaded at: www.mdpi.com/article/10.3390/insects13070626/s1, Figure S1: Map displaying the geographic origin of the *Spodoptera frugiperda* populations sampled across seasons; Figure S2: Phenotyping of diamide resistance in Brazilian *Spodoptera frugiperda* across seasons. Efficacy of a discriminating rate of flubendiamide (diet-overlay assay, 0.41 μg a.i./cm2) against 70 field populations of fall armyworm collected in 2019 (A), 2020 (B), 2021 (C), and a summary across seasons (D). Data are mean values ± CI95% (*n* = 4); Figure S3: Representative Sanger sequencing traces of the amplified PCR products for genotyping respective mutations at ryanodine receptor (RyR) position I4790 (*P. xylostella* RyR numbering; GenBank AET09964) in individual adults of flubendiamide-selected *Spodoptera frugiperda* strains BA-R and TF-R. The position of the I4790 mutation site is boxed. In strain BA-R, only one genotype was detected, I4790M; whereas in TF-R, three genotypes were found, M4790 homozygotes, K4790 homozygotes and K/M4790 heterozygotes; Figure S4: Insecticide use for lepidopteran pest control in different crops in Brazil. Number of insecticide sprays (2015–2021) and market share of important chemical classes of insecticides (2016–2021) used to manage lepidopteran larvae across states and seasons in Brazilian soybean (A), cotton (B) and corn (C). Data were obtained from BIP Spark (maize 2015–2018, soybean 2015–2021, cotton 2015–2018, 2020–2021) and Kynetec (maize 2019–2021; cotton 2019); Table S1: List of populations used for phenotypic and PCR-based allelic discrimination monitoring of *Spodoptera frugiperda* populations sampled from season 2018/19 to 2020/21; Table S2: List of populations used for genotypic (F2 Screen) of *Spodoptera frugiperda* populations sampled from season 2018/19 to 2020/21; Table S3: List of primers and probes used for fluorescent PCR-based allelic discrimination assay; Table S4: Genotyping of the frequency of RyR resistance alleles using a PCR-based assay in field populations of *Spodoptera frugiperda* sampled across seasons between 2019 and 2021. 4790 I/I, homozygous susceptible; 4790 M/M, homozygous resistant; 4790I/M and 4790 M/K, heterozygotes.
