# Supplementary material for: Large-Scale Monitoring of the Frequency of Ryanodine Receptor Target-Site Mutations Conferring Diamide Resistance in Brazilian Field Populations of Fall Armyworm, Spodoptera frugiperda (Lepidoptera: Noctuidae)
Source: Insects. 2022 Jul 13;13(7):626. doi: 10.3390/insects13070626 (PMC9323691; doi:10.3390/insects13070626)

Supporting material

Large-scale monitoring of the frequency of ryanodine receptor target-site mutations conferring diamide resistance in Brazilian field populations of fall armyworm, *Spodoptera frugiperda* (Lepidoptera: Noctuidae)

Daniela M. Okuma [a,b], Ana Cuenca [b], Ralf Nauen [c,*], Celso Omoto [a,*]

[a] *Department of Entomology and Acarology, University of São Paulo (ESALQ/ USP), Piracicaba, São Paulo, Brazil.*
[b] *Bayer SA, Crop Science Division, Paulinia, Brazil*
[c] *Bayer AG, Crop Science Division, R&D Pest Control, Monheim, Germany*

*Corresponding authors: ralf.nauen@bayer.com, celso.omoto@usp.br

Table of contents

Table S1. List of populations used for phenotypic and PCR-based allelic discrimination monitoring of *Spodoptera frugiperda* populations sampled from season 2018/19 to 2020/21.

Table S2. List of populations used for genotypic (F_2 Screen) of *Spodoptera frugiperda* populations sampled from season 2018/19 to 2020/21.

Table S3. List of primers and probes used for fluorescent PCR-based allelic discrimination assay.

Table S4. Genotyping of the frequency of RyR resistance alleles using a PCR-based assay in field populations of *Spodoptera frugiperda* sampled across seasons between 2019 and 2021. 4790 I/I, homozygous susceptible;4790 M/M, homozygous resistant; 4790I/M and 4790 M/K, heterozygotes.

Figure S1. Map displaying the geographic origin of the *Spodoptera frugiperda* populations sampled across seasons.

Figure S2. Phenotyping of diamide resistance in Brazilian *Spodoptera frugiperda* across seasons. Efficacy of a discriminating rate of flubendiamide (diet-overlay assay, 0.41 µg a.i./cm2) against 70 field populations of fall armyworm collected in 2019 (A), 2020 (B), 2021 (C), and a summary across seasons (D). Data are mean values ± CI95% (n=4).

Figure S3. Representative Sanger sequencing traces of the amplified PCR products for genotyping respective mutations at ryanodine receptor (RyR) position I4790 (P. xylostella RyR numbering; GenBank AET09964) in individual adults of flubendiamide-selected Spodoptera frugiperda strains BA-R and TF-R. The position of the I4790 mutation site is boxed. In strain BAR
only one genotype was detected, I4790M. Whereas in TF-R three genotypes were found, M4790 homozygotes, K4790 homozygotes and K/M4790 heterozygotes.

Figure S4. Insecticide use for lepidopteran pest control in different crops in Brazil. Number of insecticide sprays (2015-2021) and market share of important chemical classes of insecticides (2016-2021) used to manage lepidopteran larvae across states and seasons in Brazilian soybean (A), cotton (B) and corn (C). Data were obtained from BIP Spark (maize 2015-2018, soybean 2015-2021, cotton 2015-2018, 2020-2021) and Kynetec (maize 2019-2021; cotton 2019).

Table S1. List of populations used for phenotypic and PCR-based allelic discrimination monitoring of *Spodoptera frugiperda* populations sampled from season 2018/19 to 2020/21.

Year	Location	Season	Crop	Latitude	Longitude
2018/19	Santo Ângelo, RS	1st	Maize	28º 06’ 15.59” S	54º 25’ 33.20” W
2018/19	Selbach, SC	1st	Maize	28º 36’ 46.93” S	52º 56’ 36.06” W
2018/19	Toledo, PR	1st	Maize	24º 52’ 51.70” S	53º 35’ 46.07” W
2018/19	Ponta Grossa, PR	1st	Maize	24º 35’ 5.87” S	49º 41’ 47.96” W
2018/19	Cristalina, GO	1st	Maize	16º 58’ 49.76” S	47º 38’ 28.33” W
2018/19	Santa Helena de Goiás, GO	1st	Maize	17º 40’ 21.56” S	50º 39’ 20.32” W
2018/19	Chapecó, SC	1st	Maize	27º 11’ 39.01” S	52º 39’ 32.00” W
2018/19	Luis E. Magalhães, BA	1st	Maize	11º 38’ 55.13” S	45º 31’ 1.10” W
2018/19	Araguari, MG	1st	Maize	18º 33’ 47.52” S	47º 25’ 12.90” W
2018/19	Casa Branca, SP	1st	Maize	21º 32’ 5.49” S	47º 04’ 28.92” W
2018/19	Toledo, PR	2nd	Maize	24º 38’ 52.27” S	53º 42’ 39.65” W
2018/19	Campo Mourão, PR	2nd	Maize	24º 01’ 41.67” S	52º 19’ 5.56” W
2018/19	Casa Branca, SP	2nd	Maize	21º 45’ 0.69” S	47º 8’ 9.39” W
2018/19	Araguari, MG	2nd	Maize	18º 33’ 47.52” S	47º 25’ 12.90” W
2018/19	Chapadão do Sul, MS	2nd	Maize	18º 44’ 13.96” S	52º 32’ 8.74” W
2018/19	Lucas do Rio Verde, MT	2nd	Maize	12º 55’ 36.64” S	56º 03’ 13.73” W
2018/19	Campo Verde, MT	2nd	Maize	15º 23’ 14.16” S	55º 05’ 46.11” W
2018/19	Cristalina, GO	2nd	Maize	17º 48’ 24.11” S	50º 48’ 12.13” W
2018/19	Rio Verde, GO	2nd	Maize	17º 48’ 24.11” S	50º 48’ 12.13” W
2018/19	Casa Branca, SP	Off-season	Maize	21º 54’ 43.75” S	47º 08’ 29.39” W
2018/19	Lucas do Rio Verde, MT	Off-season	Maize	12º 58’ 44.45” S	55º 52’ 24.91” W
2018/19	Rio Verde, GO	Off-season	Maize	17º 45’ 5.85” S	50º 32’ 52.03” W
2018/19	Correntina, BA	Off-season	Maize	13º 23’ 57.23” S	45º 52’ 50.09” W
2019/20	Coronel Barros, RS	1st	Maize	28°22’54.59’’ S	54°00’52.56’’ W
2019/20	Selbach, RS	1st	Maize	28°38’45.28’’ S	53°00’17.50’’ W
2019/20	Chapecó, SC	1st	Maize	27°11’38.11’’ S	52°37’31.86’’ W
2019/20	Corbélia, PR	1st	Maize	24°50’46.36’’ S	53°19’10.86’’ W
2019/20	Tibagi, PR	1st	Maize	24°27’9.86’’ S	50°11’24.90’’ W
2019/20	Pirassununga, SP	1st	Sorghum	21°53’56.01’’ S	47°21’10.87’’ W
2019/20	Uberlândia, MG	1st	Sorghum	19°10’49.32’’ S	48°09’16.56’’ W
2019/20	Santa Helena de Goiás, GO	1st	Sorghum	17°45’13.68’’ S	50°35’12.19’’ W
2019/20	Cristalina, GO	1st	Maize	16°27’07.21’’ S	47°37’48.60’’ W
2019/20	Casa Branca, SP	2nd	Maize	21°54’45.2’’ S	47°10’52.52’’ W
2019/20	Londrina, PR	2nd	Maize	23°20.26’26.9’’ S	51°14’04.6’’ W
2019/20	Cascavel, PR	2nd	Maize	24°36’26.2’’ S	53°17’40.6’’ W
2019/20	Mamborê, PR	2nd	Maize	24°19’04.80’’ S	52°35’23.7’’ W
2019/20	Dourados, MS	2nd	Maize	22°03’43’’ S	54°54’37’’ W
2019/20	Chapadão do Sul, MS	2nd	Maize	18°42’57’’ S	52°45’32’’ W
2019/20	Lucas do Rio Verde, MT	2nd	Maize	12°59’36.27’’ S	55°50’06.40’’ W
2019/20	Sapezal, MT	2nd	Maize	12°26’54.42’’ S	59°15’06.33’’ W
2019/20	Campo Verde, MT	2nd	Maize	15°32’40.28’’ S	55°12’09.48’’ W
2019/20	Rio Verde, GO	2nd	Maize	17°48’03.43’’ S	50°24’59.66’’ W
2019/20	Campo Novo do Parecis, MT	Off-season	Cotton	11°42’56.16’’ S	55°18’44.74’’ W
2019/20	Campo Verde, MT	Off-season	Cotton	15º33’53.90” S	55º11’27.20“ W
2019/20	Roda Velha, BA	Off-season	Cotton	12º40’33.56” S	45º58’00.90” W
2019/20	Luis E. Magalhães, BA	Off-season	Cotton	11º41’47.28” S	46º00’17.46“ W
2019/20	Jaborandi, BA	Off-season	Cotton	14º14’33.36” S	45º26’32.52” W

2020/21	Santo Ângelo, RS	1st	Maize	28°28’50.40’’ S	54°20’16.90’’ W
2020/21	Chapecó, SC	1st	Maize	27°05’19’’ S	52°38’13’’ W
2020/21	Selbach, SC	1st	Maize	28°38’33.70’’ S	52°57’05.20’’ W
2020/21	Cascavel, PR	1st	Maize	25°02’12.48’’ S	53°04’12.61’’ W
2020/21	Piraí do Sul, PR	1st	Maize	24°27’40.84’’ S	50°10’38.84’’ W
2020/21	Casa Branca, SP	1st	Maize	21°48’05.46’’ S	46°55’54.99’’ W
2020/21	Araguari, MG	1st	Maize	18°43’54.43’’ S	47°58’41.06’’ W
2020/21	Santa Helena de Goiás, GO	1st	Maize	17°41’21.17’’ S	50°39’09.68’’ W
2020/21	Cristalina, GO	1st	Maize	16°28’08.58’’ S	47°39’11.72’’ W
2020/21	Luis E. Magalhães, BA	1st	Maize	12°05’40.82’’ S	45°42’41.54’’ W
2020/21	Campo Mourão, PR	2nd	Maize	24°02’11.52’’ S	52°18’37.14’’ W
2020/21	Londrina, PR	2nd	Maize	23°19’46.21’’ S	51°18’19.93’’ W
2020/21	Cascavel, PR	2nd	Maize	24°21’51.21’’ S	53°33’38.10’’ W
2020/21	Dourados, MS	2nd	Maize	22°11’49.02’’ S	54°42’42.98’’ W
2020/21	Chapadão do Sul, MS	2nd	Maize	18°43’55.58’’ S	52°30’48.33’’ W
2020/21	Lucas do Rio Verde, MT	2nd	Maize	13°01’51.74’’ S	55°53’52.28’’ W
2020/21	Sapezal, MT	2nd	Maize	13°28’52.18’’ S	59°16’16.98’’ W
2020/21	Campo Verde, MT	2nd	Maize	15°15’13.70’’ S	55°02’23.27’’ W
2020/21	Rio Verde, GO	2nd	Maize	17°48’03.43’’ S	50°24’59.66’’ W
2020/21	São Desidério, BA	Off-season	Cotton	12º 40’33.56” S	45º.58’00.90” W

Table S2. List of populations used for genotypic (F_2 Screen) of *Spodoptera frugiperda* populations sampled from season 2018/19 to 2020/21.

Season	Location	Crop	Latitude	Longitude	Resistance allele frequency	95% IC
2018/19	Tasso Fragoso, MA	Maize	7°49'33.8" S	46°00'22.6" W	0.0430	0.0248 - 0.0658
2018/19	Luis Eduardo Magalhães, BA	Maize	13°19'31.6“ S	46°05'57.3 W	0.1023	0.0729 - 0.1359
2018/19	Campo Verde, MT	Maize	15°30'53“ S	55°12'25" W	0.0485	0.0317 - 0.0685
2018/19	Lucas do Rio Verde, MT	Maize	12°48'15“ S	56°25'40“ W	0.0240	0.0116 - 0.0408
2018/19	Chapadão do Sul, MS	Maize	18°42'52.6"S	52°36'23.5" W	0.0167	0.0021 - 0.0460
2018/19	Ponta Porã, MS	Maize	22°24'50.9"S	55°19'18.4" W	0.0119	0.0015 - 0.0330
2018/19	Rio Brilhante, MS	Maize	21°42'37.4“ S	54°39'09.8“ W	0.0482	0.0279 - 0.0734
2018/19	Rio Verde, GO	Maize	17°48'49.8" S	50°39'30.2" W	0.0489	0.0317 - 0.0685
2018/19	Araguari, MG	Maize	18°40'23.9" S	48°09'27.1" W	0.0606	0.0352 - 0.0924
2018/19	Casa Branca, SP	Maize	21°50'03.8" S	46°58'50.7" W	0.0121	0.0045 - 0.0234
2018/19	Campo Mourão, PR	Maize	23°45‘24.908“ S	52°13‘10.902“	0.0396	0.0228 - 0.0606
2018/19	Londrina, PR	Maize	22°55‘35.501“ S	50°54‘ 3.6“ W	0.0577	0.0328 - 0.0891
2019/20	Serra do Coceiral, BA	Maize	10°37′53,541″ S	45°45′ 59,756″ W	0.0635	0.03755 - 0.09553
2019/20	Campo Verde, MT	Maize	15°38′25,736″ S	55°14′44,977″ W	0.0210	0.00056 - 0.07588
2019/20	Lucas do Rio Verde, MT	Maize	13°17′24,515″ S	55°38′50,917″ W	0.0068	0.00018 - 0.024919
2019/20	Maracaju, MS	Maize	22°9′32,573″ S	54°38′42,594″ W	0.0485	0.01816 - 0.09241
2019/20	Ponta Porã, MS	Maize	22°27′8,042″ S	55°20′33,054″ W	0.0211	0.00970 - 0.03661
2019/20	Formosa, GO	Maize	14°55′46,255″ S	47°35′54,942″ W	0.0345	0.01510 - 0.06149
2019/20	Araguari, MG	Maize	23º45’24.9’’ S	48º09’27.10’’ W	0.0630	0.00176 - 0.21892
2019/20	Casa Branca, SP	Maize	21°48′18,093″ S	47°10′57,058″ W	0.1112	0.05994 - 0.17658
2019/20	Campo Mourão, PR	Maize	24°19’04,80’’ S	52°35’23,7’’ W	0.0098	0.00270 - 0.02142
2019/20	Londrina, PR	Maize	22°59′31,725″ S	51°0′34,043″ W	0.0070	0.00018 - 0.02561
2020/21	Balsas, MA	Maize	7º49’33.8’’ S	46º00’22.6’’ W	0.0635	0.03755 - 0.09553
2020/21	Campo Verde, MT	Maize	15º30’53.0’’ S	55º12’25.0’’ W	0.0210	0.00056 - 0.07588
2020/21	Lucas do Rio Verde, MT	Maize	12º48’15.0’’ S	56º25’40.0’’ W	0.0068	0.00018 - 0.024919
2020/21	Maracaju, MS	Maize	22º55’35.5’’ S	54º39’09.8’’ W	0.0485	0.01816 - 0.09241
2020/21	Ponta Porã, MS	Maize	22º24’50.9’’ S	55º19’18.4’’ W	0.0211	0.00970 - 0.03661
2020/21	Rio Verde, GO	Maize	17º48’49.8’’ S	50º39’30.2’’ W	0.0345	0.01510 - 0.06149
2020/21	Araguari, MG	Maize	23º45’24.9’’ S	48º09’27.10’’ W	0.0630	0.00176 - 0.21892
2020/21	Campo Mourão, PR	Maize	18º40’23.9’’ S	52º13’10.9’’ W	0.1112	0.05994 - 0.17658
2020/21	Londrina, PR	Maize	21º42’37’’ S	50º54’03.6’’ W	0.0098	0.00270 - 0.02142

Table S3. List of primers and probes used for PCR-based allelic discrimination assays

Primer	Sequence
Sf.taq_4790_F	5'-ACGACGATGCACTAGAAG-3'
Sf.taq_4790_R	5'-CACCTTGAGATGATAGTACC-3'
Sf.4790I_sus_FAM	5’-[FAM]TGTCGCTCGCTATACTCATCG[BHQ1]-3'
Sf.4790M_mut_VIC	5’-[6VIC]CTCGCTATGCTCATCGGGT[BHQ1]-3'
Sf.4790K_mut_VIC	5'-[6VIC]TGCTCGCTAAACTCATCGGGT[BHQ1]-3'
Sf. 1-F	5'-TCAAGGTGGCTGCAGTACTG-3'
Sf. 2-F	5'-GCCATCGAAGCTGAGAGCAA-3'
Sf. 3-F	5'-AGGCAGCAAGGGACTGATTC-3'
Sf. 1-R	5'-GTTCCTGTTGACCTCGTCGT-3'
Sf. 2-R	5'-GAAGAAGTCCCAGCATCGCT-3'

Table S4. Genotyping of the frequency of RyR resistance alleles using a PCR-based assay in field populations of *Spodoptera frugiperda* sampled across seasons between

2019 and 2021. 4790 I/I, homozygous susceptible; 4790 M/M and K/K, homozygous resistant; 4790 I/M, 4790 I/K and 4790 M/K, heterozygotes.

Year	City	State	Season	N	4790 I/I	4790 I/M	4790 M/M	4790 M/K	F	X^2	P-value	4790 I/I	4790 I/K	4790 K/K	4790 M/K	F	X^2	P-value
2019	Selbach	RS	1st	20	100%	0%	0%	0%	0,00	0,00	1,00	100%	0%	0%	0%	0,00	0,00	1,00
2019	Santo Ângelo	RS	1st	18	100%	0%	0%	0%	0,00	0,00	1,00	100%	0%	0%	0%	0,00	0,00	1,00
2019	Toledo	PR	1st	19	90%	10%	0%	0%	0,05	0,05	0,82	90%	0%	0%	0%	0,00	0,19	0,66
2019	Ponta Grossa	PR	1st	18	100%	0%	0%	0%	0,00	0,00	1,00	100%	0%	0%	0%	0,00	0,00	1,00
2019	Cristalina	GO	1st	8	100%	0%	0%	0%	0,00	0,00	1,00	100%	0%	0%	0%	0,00	0,00	1,00
2019	Luis Eduardo Magalhães	BA	1st	17	71%	24%	6%	0%	0,18	0,62	0,43	71%	0%	0%	0%	0,00	1,47	0,23
2019	Chapecó	SC	1st	16	100%	0%	0%	0%	0,00	0,00	1,00	100%	0%	0%	0%	0,00	0,00	1,00
2019	Santa Helena de Goiás	GO	1st	18	83%	17%	0%	0%	0,08	0,15	0,70	83%	0%	0%	0%	0,00	0,50	0,48
2019	Araguari	MG	1st	10	90%	10%	0%	0%	0,05	0,03	0,87	90%	0%	0%	0%	0,00	0,10	0,75
2019	Casa Branca	SP	1st	19	95%	5%	0%	0%	0,03	0,01	0,91	95%	0%	0%	0%	0,00	0,05	0,83
2019	Toledo	PR	2nd	19	100%	0%	0%	0%	0,00	0,00	1,00	100%	0%	0%	0%	0,00	0,00	1,00
2019	Lucas do Rio Verde	MT	2nd	19	85%	15%	0%	0%	0,08	0,12	0,72	85%	0%	0%	0%	0,00	0,43	0,51
2019	Sapezal	MT	2nd	20	85%	15%	0%	0%	0,08	0,13	0,72	85%	0%	0%	0%	0,00	0,45	0,50
2019	Campo Mourão	PR	2nd	19	100%	0%	0%	0%	0,00	0,00	1,00	100%	0%	0%	0%	0,00	0,00	1,00
2019	Londrina	PR	2nd	20	100%	0%	0%	0%	0,00	0,00	1,00	100%	0%	0%	0%	0,00	0,00	1,00
2019	Chapadão do Sul	MS	2nd	20	80%	20%	0%	0%	0,10	0,25	0,62	80%	0%	0%	0%	0,00	0,80	0,37
2019	Dourados	MS	2nd	20	75%	20%	5%	0%	0,15	0,93	0,33	75%	0%	0%	0%	0,00	1,25	0,26
2019	Casa Branca	SP	2nd	20	75%	25%	0%	0%	0,13	0,41	0,52	75%	0%	0%	0%	0,00	1,25	0,26
2019	Campo Verde	MT	2nd	18	94%	6%	0%	0%	0,03	0,01	0,90	94%	0%	0%	0%	0,00	0,06	0,81
2019	Rio Verde	GO	2nd	20	80%	20%	0%	0%	0,10	0,25	0,62	80%	0%	0%	0%	0,00	0,80	0,37
2019	Correntina	BA	Off-season	19	68%	16%	10%	0%	0,19	3,53	0,06	68%	5%	0%	0%	0,04	1,38	0,24
2019	Santa Helena de Goiás	GO	Off-season	17	82%	18%	0%	0%	0,09	0,16	0,69	82%	0%	0%	0%	0,00	0,53	0,47
2019	Lucas do Rio Verde	MT	Off-season	20	95%	5%	0%	0%	0,03	0,01	0,91	95%	0%	0%	0%	0,00	0,05	0,82
2019	Chapadão do Sul	MS	Off-season	20	95%	5%	0%	0%	0,03	0,01	0,91	95%	0%	0%	0%	0,00	0,05	0,82
2020	Santo Ângelo	RS	1st	20	100%	0%	0%	0%	0,00	0,00	1,00	100%	0%	0%	0%	0,00	0,00	1,00
2020	Selbach	RS	1st	20	100%	0%	0%	0%	0,00	0,00	1,00	100%	0%	0%	0%	0,00	0,00	1,00
2020	Pirassununga	SP	1st	20	95%	5%	0%	0%	0,03	0,01	0,91	95%	0%	0%	0%	0,00	0,05	0,82
2020	Tibagi	PR	1st	20	90%	10%	0%	0%	0,05	0,06	0,81	90%	0%	0%	0%	0,00	0,20	0,65
2020	Cascavel	PR	1st	21	100%	0%	0%	0%	0,00	0,00	1,00	100%	0%	0%	0%	0,00	0,00	1,00
2020	Chapecó	SC	1st	6	100%	0%	0%	0%	0,00	0,00	1,00	100%	0%	0%	0%	0,00	0,00	1,00
2020	Cristalina	GO	1st	10	100%	0%	0%	0%	0,00	0,00	1,00	100%	0%	0%	0%	0,00	0,00	1,00
2020	Uberlândia	MG	1st	20	95%	5%	0%	0%	0,03	0,01	0,91	95%	0%	0%	0%	0,00	0,05	0,82
2020	Luis Eduardo Magalhães	BA	1st	21	90%	0%	0%	0%	0,00	0,21	0,65	90%	5%	5%	0%	0,08	8,59	0,00
2020	Lucas do Rio Verde	MT	2nd	20	90%	10%	0%	0%	0,05	0,06	0,81	90%	0%	0%	0%	0,00	0,20	0,65
2020	Campo Verde	MT	2nd	19	100%	0%	0%	0%	0,00	0,00	1,00	100%	0%	0%	0%	0,00	0,00	1,00
2020	Casa Branca	SP	2nd	15	60%	27%	7%	0%	0,21	0,37	0,54	60%	7%	0%	0%	0,05	1,69	0,19
2020	Sapezal	MT	2nd	17	100%	0%	0%	0%	0,00	0,00	1,00	100%	0%	0%	0%	0,00	0,00	1,00
2020	Londrina	PR	2nd	20	100%	0%	0%	0%	0,00	0,00	1,00	100%	0%	0%	0%	0,00	0,00	1,00
2020	Cascavel	PR	2nd	8	75%	13%	13%	0%	0,19	2,78	0,10	75%	0%	0%	0%	0,00	0,50	0,48
2020	Chapadão do Sul	MS	2nd	21	100%	0%	0%	0%	0,00	0,00	1,00	100%	0%	0%	0%	0,00	0,00	1,00
2020	Campo Novo do Parecis	MT	Off-season	20	75%	15%	10%	0%	0,18	4,62	0,03	75%	0%	0%	0%	0,00	1,25	0,26

2020	Barreiras	BA	Off-season	19	84%	11%	5%	0%	0,11	3,70	0,05	84%	0%	0%	0%	0,00	0,47	0,49
2020	Campo Verde	MT	Off-season	21	90%	10%	0%	0%	0,05	0,05	0,82	90%	0%	0%	0%	0,00	0,19	0,66
2020	Jaborandi	BA	Off-season	14	86%	14%	0%	0%	0,07	0,08	0,77	86%	0%	0%	0%	0,00	0,29	0,59
2021	Selbach	RS	1st	20	100%	0%	0%	0%	0,00	0,00	1,00	100%	0%	0%	0%	0,00	0,00	1,00
2021	Santo Ângelo	RS	1st	18	100%	0%	0%	0%	0,00	0,00	1,00	100%	0%	0%	0%	0,00	0,00	1,00
2021	Casa Branca	SP	1st	20	95%	0%	0%	0%	0,00	0,05	0,82	95%	5%	0%	0%	0,03	0,01	0,91
2021	Santa Helena de Goiás	GO	1st	20	95%	5%	0%	0%	0,03	0,01	0,91	95%	0%	0%	0%	0,00	0,05	0,82
2021	Ponta Grossa	PR	1st	20	100%	0%	0%	0%	0,00	0,00	1,00	100%	0%	0%	0%	0,00	0,00	1,00
2021	Cascavel	PR	1st	20	100%	0%	0%	0%	0,00	0,00	1,00	100%	0%	0%	0%	0,00	0,00	1,00
2021	Chapecó	SC	1st	20	100%	0%	0%	0%	0,00	0,00	1,00	100%	0%	0%	0%	0,00	0,00	1,00
2021	Araguari	MG	1st	20	90%	5%	5%	0%	0,08	8,18	0,00	90%	0%	0%	0%	0,00	0,20	0,65
2021	Luis Eduardo Magalhães	BA	1st	20	70%	10%	10%	0%	0,17	6,03	0,01	70%	10%	0%	0%	0,06	0,86	0,35
2021	Chapadão do Sul	MS	2nd	20	90%	0%	10%	0%	0,10	20,00	0,00	90%	0%	0%	0%	0,00	0,20	0,65
2021	Dourados	MS	2nd	20	100%	0%	0%	0%	0,00	0,00	1,00	100%	0%	0%	0%	0,00	0,00	1,00
2021	Campo Mourão	PR	2nd	20	100%	0%	0%	0%	0,00	0,00	1,00	100%	0%	0%	0%	0,00	0,00	1,00
2021	Rio Verde	GO	2nd	20	85%	10%	5%	0%	0,10	3,95	0,05	85%	0%	0%	0%	0,00	0,45	0,50
2021	Casa Branca	SP	2nd	20	90%	0%	0%	0%	0,00	0,20	0,65	90%	10%	0%	0%	0,05	0,06	0,81
2021	Lucas do Rio Verde	MT	2nd	20	80%	15%	5%	0%	0,13	1,98	0,16	80%	0%	0%	0%	0,00	0,80	0,37
2021	Sapezal	MT	2nd	20	90%	5%	5%	0%	0,08	8,18	0,00	90%	0%	0%	0%	0,00	0,20	0,65
2021	Campo Verde	MT	2nd	20	80%	5%	10%	0%	0,13	10,74	0,00	80%	0%	5%	0%	0,06	2,45	0,12
2021	Cascavel	PR	2nd	20	95%	5%	0%	0%	0,03	0,01	0,91	95%	0%	0%	0%	0,00	0,05	0,82
2021	Londrina	PR	2nd	19	100%	0%	0%	0%	0,00	0,00	1,00	100%	0%	0%	0%	0,00	0,00	1,00
2021	São Desidério	BA	Off-season	19	42%	26%	20%	0%	0,38	2,23	0,13	42%	11%	0%	0%	0,10	4,33	0,04

Figure S1. Map displaying the geographic origin of the FAW populations sampled across seasons.

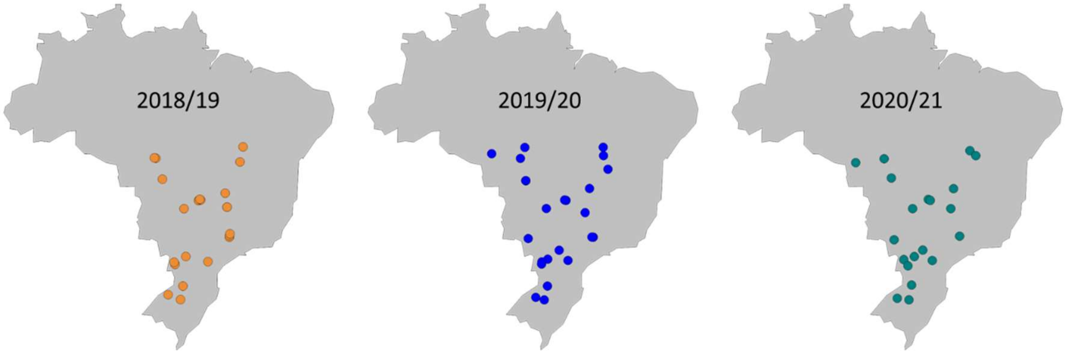

Figure S2. Phenotyping of diamide resistance in Brazilian *Spodoptera frugiperda* across seasons. Efficacy of a discriminating rate of flubendiamide (diet-overlay assay, 0.41 µg a.i./cm2) against field populations of fall armyworm collected in 2019 (A), 2020 (B), 2021 (C), and a summary across seasons (D). Data are mean values ± CI95% (n=4).

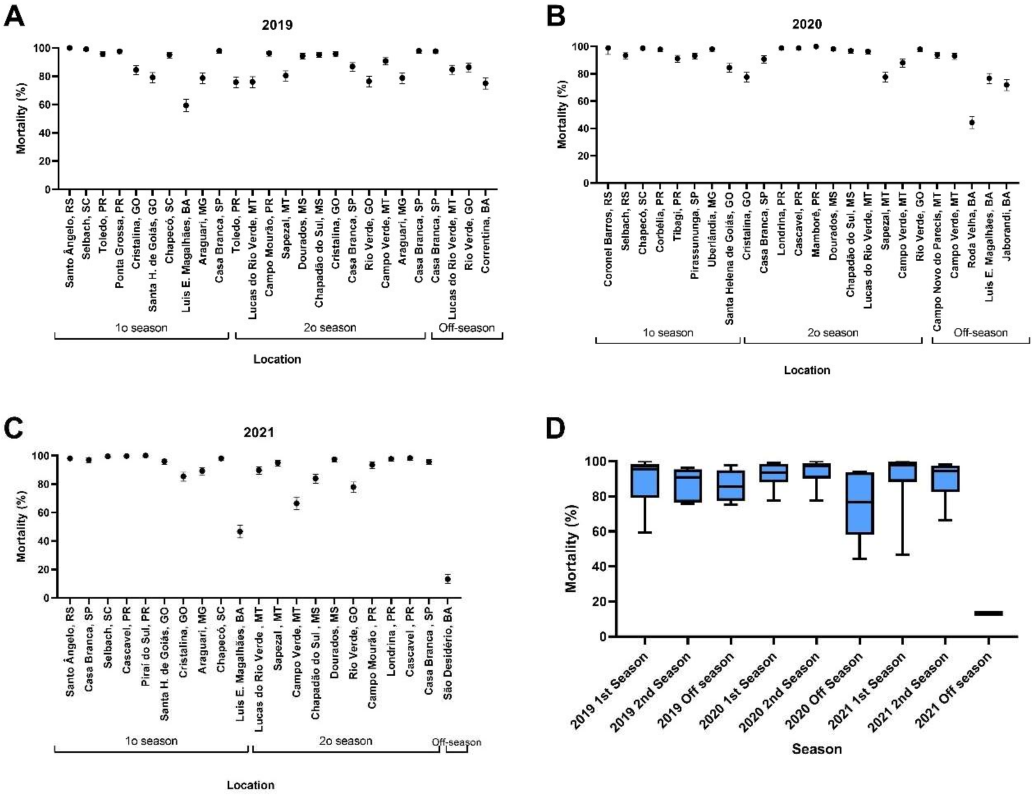

Figure S3. Representative Sanger sequencing traces of the amplified PCR products for genotyping respective mutations at ryanodine receptor (RyR) position I4790 (*P. xylostella* RyR numbering; GenBank AET09964) in individual adults of flubendiamide-selected *Spodoptera frugiperda* strains BA-R and TF-R. The position of the I4790 mutation site is boxed. In strain BA-R only one genotype was detected, I4790M. Whereas in TF-R three genotypes were found, M4790 homozygotes, K4790 homozygotes and K/M4790 heterozygotes.

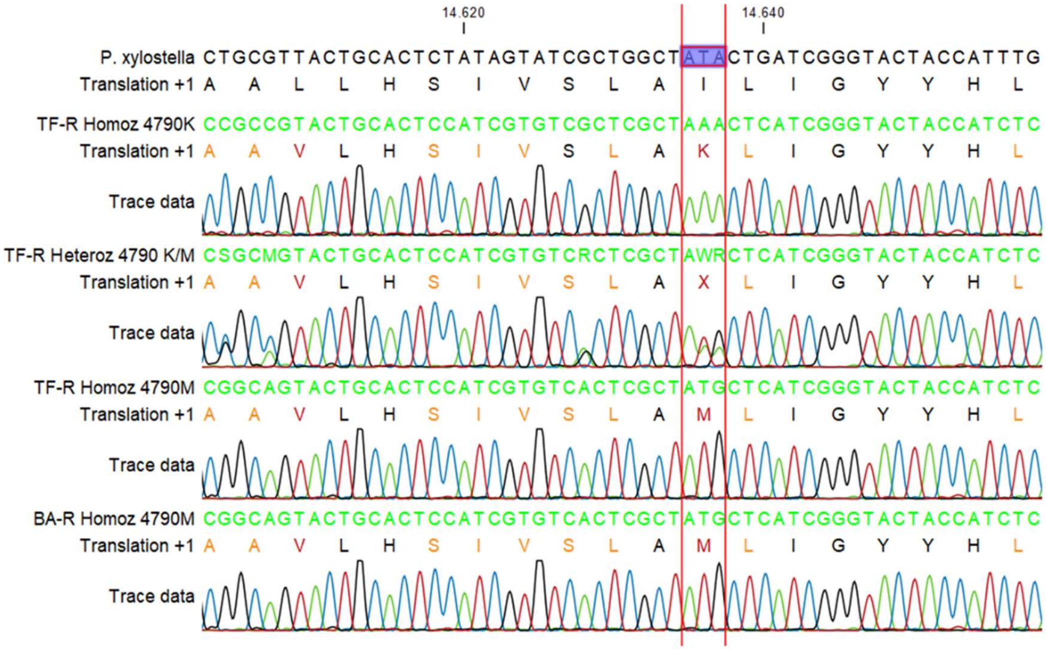

Figure S4. Insecticide use for lepidopteran pest control in different crops in Brazil. Number of insecticide sprays (2015-2021) and market share of important chemical classes of insecticides (2016-2021) used to manage lepidopteran larvae across states and seasons in Brazilian soybean (A), cotton (B) and corn (C). Data were obtained from BIP Spark (maize 2015-2018, soybean 2015-2021, cotton 2015-2018, 2020-2021) and Kynetec (maize 2019-2021; cotton 2019).

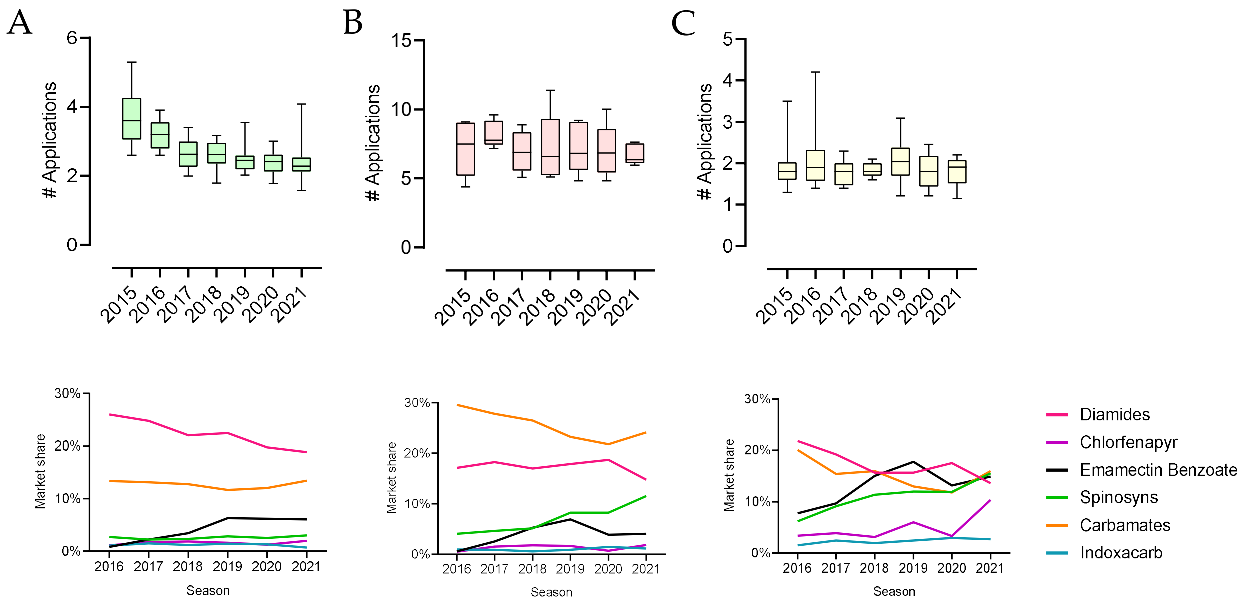

Supplement: Supplementary file 1 [file insects-13-00626-s001.zip › insects-1803004-supplementary.pdf]
